# Squamous Cell Carcinoma of the Liver Originating from a Solitary Non-Parasitic Cyst: Case Report and Review of the Literature

**DOI:** 10.1155/1996/97680

**Published:** 1996

**Authors:** Arved Weimann, Jürgen Klempnauer, Michael Gebel, Hansjörg Maschek, Michael Bartels, Burckhardt Ringe, Rudolf Pichlmayr

**Affiliations:** 1 Abteilung Gastroenterologie Medizinische Hochschule Hannover Hannover Germany; 2 Institut für Pathologie Medizinische Hochschule Hannover Hannover Germany; 3 Klinik für Abdominal- und Transplantationschirurgie Medizinische Hochschule Hannover Hannover Germany

## Abstract

Squamous cell carcinoma of the liver arising from a non-parasitic cyst is a rare entity of a primary liver tumor with an unfavourable prognosis. We report a case of a patient with a cyst in the right lobe leading to upper abdominal symptoms and respiratory discomfort. Malignancy was not suspected from the clinical findings or repeated cytological examination of the cyst fluid. However, the blood stained brown color of the cyst fluid was unusual. Cyst recurrence after six attempts of conservative treatment with sonography guided drainage over a period for more than one year led to laparotomy with cyst unroofing. Because frozen section from the cyst wall revealed the unexpected finding of squamous cell carcinoma right hemihepatectomy was performed during the same operation. The patient is alive more than four years after surgery without cyst or tumor recurrence. The difficulties in establishing diagnosis are confirmed by the review ofother reports. In the diagnosis and treatment ofsymptomatic non-parasitic liver cysts possible malignancy has to be considered. In case of proven carcinoma radical surgery with partial hepatectomy should be performed.


*HPB Surgery*, 1996, Vol.10, pp.45–49
Reprints available directly from the publisher
Photocopying permitted by license only

© 1996 OPA (Overseas Publishers Association)
Amsterdam B.V. Published in The Netherlands
by Harwood Academic Publishers GmbH
Printed in Malaysia

### CASE REPORT
### Squamous Cell Carcinoma of the Liver Originating from a Solitary Non-Parasitic Cyst Case Report and Review of the Literature

ARVED WEIMANN, JÜRGEN KLEMPNAUER, MICHAEL GEBEL*, HANSJÖRG MASCHEK**, MICHAEL BARTELS, BURCKHARDT RINGE and RUDOLF PICHLMAYR

Klinik für Abdominal- und Transplantationschirurgie, Abteilung Gastroenterologie* und Institut für Pathologie**, Medizinische Hochschule Hannover, Hannover, Germany

*(Received 24 October 1993)*

Squamous cell carcinoma of the liver arising from a non-parasitic cyst is a rare entity of a primary liver tumor with an unfavourable prognosis. We report a case of a patient with a cyst in the right lobe leading to upper abdominal symptoms and respiratory discomfort. Malignancy was not suspected from the clinical findings or repeated cytological examination of the cyst fluid. However, the blood stained brown color of the cyst fluid was unusual. Cyst recurrence after six attempts of conservative treatment with sonography guided drainage over a period for more than one year led to laparotomy with cyst unroofing. Because frozen section from the cyst wall revealed the unexpected finding of squamous cell carcinoma right hemihepatectomy was performed during the same operation. The patient is alive more than four years after surgery without cyst or tumor recurrence. The difficulties in establishing diagnosis are confirmed by the review of other reports. In the diagnosis and treatment of symptomatic non-parasitic liver cysts possible malignancy has to be considered. In case of proven carcinoma radical surgery with partial hepatectomy should be performed.

KEY WORDS: Primary liver tumors squamous cell carcinoma hepatic cyst cyst drainage liver resection

### INTRODUCTION

Squamous cell carcinoma is an extremely rare primary liver tumor arising in chronic inflammatory conditions or non-parasitic hepatic cysts. In the literature only a few cases of squamous cell carcinoma were thought to have developed from metaplastic change of the biliary epithelium in a non-parasitic hepatic cyst and predominantly discussed from a pathological point of view [1–9] (table 1).

*Correspondence to*: Arved Weimann, MD, Klinik für Abdominal- und Transplantationschirurgie, Medizinische Hochschule Hannover, Konstanty-Gutschow- Str. 8, 30625 Hannover, Germany, Phone.: 0049 511/532-6534, Fax no.: 0049 511/532-4010

### CASE REPORT

A 74 year old female presented for surgical treatment of a hepatic cyst initially in August of 1990. 10 months earlier, right upper abdominal and back pain with respiratory discomfort had led to sonography and CT scan with diagnosis of a solitary liver cyst in the right lobe, with a diameter of 12 × 10 cm. Presuming the diagnosis to be a congenital liver cyst, sonography guided drainage had been performed twice in March and May 1990. Temporary relief of symptoms had been followed by recurrence of the cyst.

At the time of presentation the patient complained again of right upper abdominal and back pain associated with respiratory discomfort. There was no jaundice, no weight loss, or recurrent elevated temperature. Beside caesarian section and hysterectomy there was no previous abdominal surgery. Physical examination revealed an adequate state of health. In the right upper abdomen a well defined mass could be palpated. Sonography showed a cyst in the right lobe of the liver with an atypical layer and hyperechoic structures on the floor consistent with old hematoma without irregular thickening or double contour of the cyst wall (figure 1). No renal or pancreatic cyst could be found. Peripheral blood cell count was without increase in leucocytes, anemia or eosinophilia. Liver function was normal, liver enzymes were within normal limits except for a moderate elevation of GGT (50 U/l), bilirubin levels were normal. Echinococcus serology was negative. From these findings diagnosis of congenital dysontogenetic liver cyst was made. Therefore, conservative treatment was attempted again.

In August 1990 the patient underwent sonography guided drainage via a pigtail catheter for two days and subsequent sclerotherapy with ethoxysclerol. Due to recurrent cyst and symptoms, the procudure had to be repeated in December 1990, March and May 1991. Every time the drained cyst fluid was brown and blood stained. Cytology revealed cell detritus, red blood cells, endothelial cells, macrophages, but no tumor cells (PAP II). Bacteriology was negative. Related to previous examinations there was no change in laboratory tests. However, erythrocyte sedimentation rate which had not been measured before was elevated (55/130 mm). Finally the cyst had a size of 17.5 × 14 × 15cm. The atypical cyst recurrence, refractory to conservative treatment led to the decision to proceed with laparotomy with surgical drainage and unroofing of the cyst.

During laparotomy on 15–7–91 the solitary cyst was found within the liver segments VI, VII and VIII. There was a remarkable adhesion to the right diaphragm but no signs of malignancy were observed. There was no lymphnode enlargement in the hepatic ligament. After careful drainage of brown fluid, the cyst roof was resected and a biopsy of the wall taken for frozen section. Histology revealed the unexpected diagnosis of a squamous cell carcinoma. For curative resection right hemihepatectomy including partial resection of the right diaphragm and cholecystectomy were performed. The postoperative course was uneventful. The patient was discharged on the 15th postoperative day. Four and a half years after surgery the patient is alive without signs of cyst or tumor recurrence.

Final histology of the resection specimen revealed a moderately differentiated partially keratinizing squamous cell carcinoma arising from the wall of a hepatic cyst (figure 2a). In the cyst wall carcinomatous

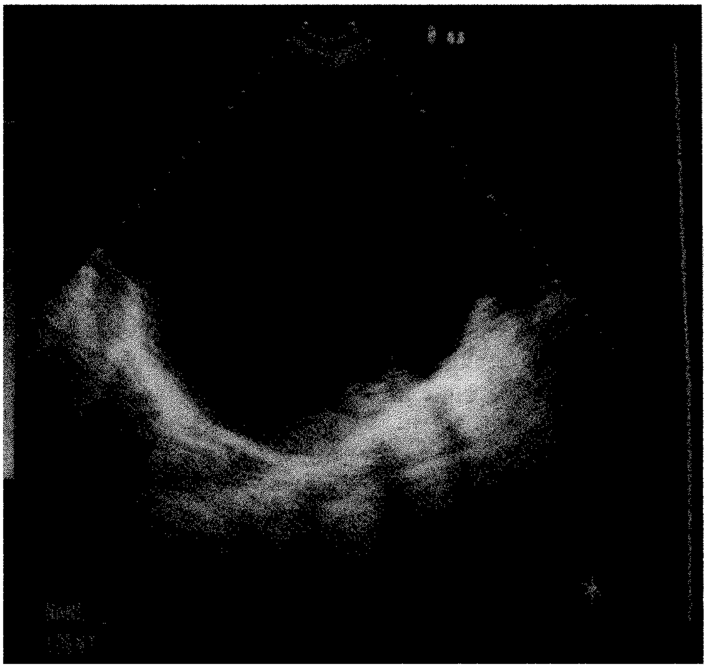

**Figure 1** Cyst in sonography with atypical layer and hyperechoic area on the floor epithelium was adjacent to sclerozing inflammation (figure 2b). There was focal infiltration of the fibrous capsule but not into liver parenchyma (figure 2a). Adjacent liver parenchyma showed slight cholangitis.

### DISCUSSION

### *Pathology*

Carcinoma deriving from benign non-parasitic hepatic cyst is extremely rare. In our review of the literature 9 cases of squamous cell carcinoma described as arising from hepatic cysts were found[1-9]. While the cyst was solitary in all other cases, Pliskin *et al.*[7] found advanced carcinoma in two of three different cysts in the same patient. Etiology is rather likely resulting from secondary squamous metaplasia due to chronic inflammation of biliary lined cyst or duct and subsequent neoplastic transformation[5]. This fits with the histology in our case with carcinomatous epithelium next to chronic inflammatory changes. There are also cases of primary adenocarcinoma originating from hepatic cysts[10-15]. Mizumoto and Kawarada[15] recommended the distinction between cystadenocarcinoma probably originating from cystadenoma[16] and carcinoma arising from hepatic cyst. However, this may be difficult in advanced cases and Azizah and Paradinas[17] argued that some of the published adenocarcinomas arising from hepatic cysts might actually have been cystadenocarcinoma. Furthermore, these authors distinguished cholangiocarcinoma coexisting with developmental nonneoplastic liver cyst. Squamous cell carcinoma of the liver can also be found without occurence of a cyst [18,19] as well as a component of intrahepatic cholangiocarcinoma the so called adenosquamous carcinoma[20,21].

### *Symptoms, clinical findings and definite diagnosis*

In our 74 years old female patient, the symptoms leading to diagnosis of a liver tumor were pain due to

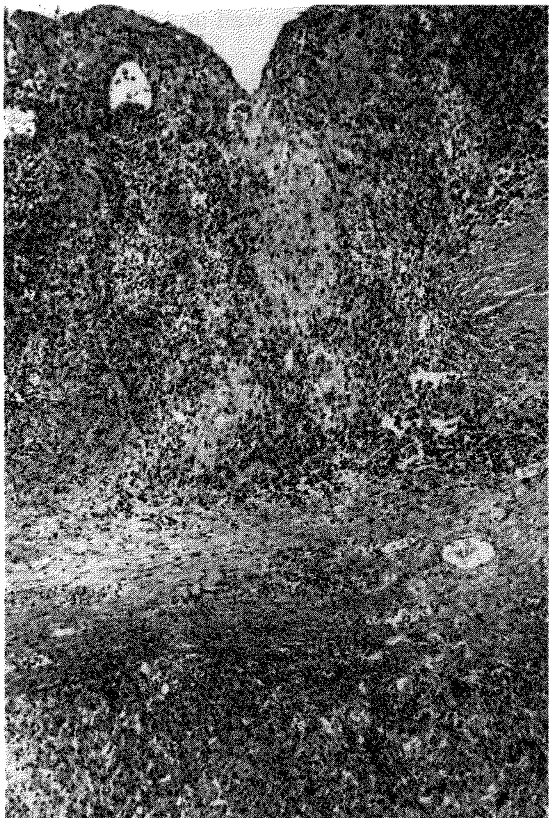

**Figure 2a** Low power view of the cyst wall with adjacent liver tissue (at the bottom). H&E, original magnification × 16

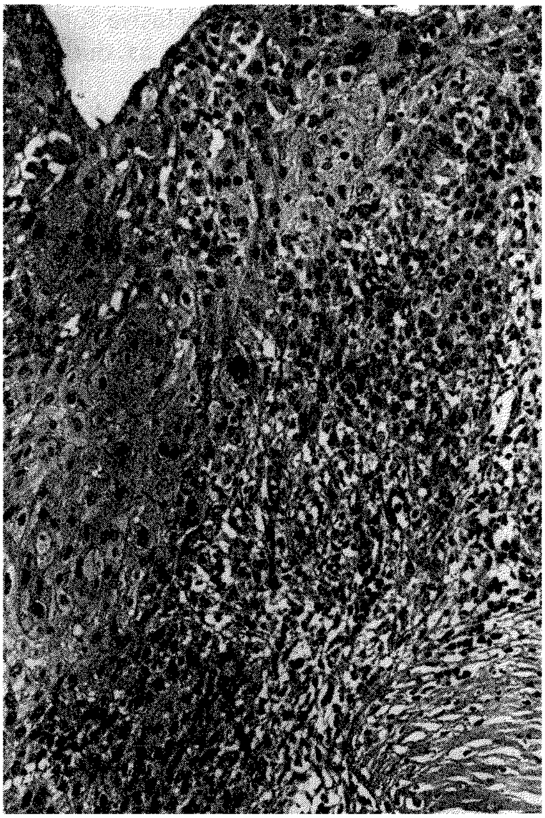

**Figure 2b** Squamous cell carcinoma arising in the inner cyst wall with chronic inflammation. Note the keratinization of squamous cells at the left. H&E, original magnification × 40

a mass in the right upper quarter of the abdomen. Back pain and respiratory discomfort, which were not accompanied by diminished pulmonary function parameters, may have been related to cyst adhesion to the diaphragm. During a period of one year, no deterioration in clinical status occured. In the other reported cases [2,3,5–9] jaundice was observed in four patients [2,5,6,8], which can be explained in three of them by the central cyst location in the hilar area with compression of the major bile ducts. One patient had dyspepsia for a long period and recent weight loss of 19 kg before admission [2], another ascites which was negative for tumor cells deriving from thrombosis of the portal vein and the hepatic veins protruded into the inferior vena cava [5]. From clinical findings, malignancy had to be expected in these two patients. In three cases[6–8]. Despite negative echinococcal serology, due to calcification of the cyst wall in [9] the findings were thought to be consistent with echinococcal cyst. CT-scan was suspicious for a malignant liver tumor – in one by chest X-ray showing bilateral nodules – even for metastatic disease. In Bloustein's[3] and our patient symptoms, primary clinical findings and diagnostic imaging were conclusive for benign solitary non-parasitic hepatic cyst as outlined by Clark *et al.*[22].

The diagnosis of squamous cell carcinoma arising from a cyst is very difficult to make. Even exploratory laparotomy with biopsy can be negative. In the cases of Bloustein[3] and Lynch[8] definite diagnosis could be established only by biopsy from the cyst wall during a second laparotomy which was performed for cyst recurrence after surgical drainage. As found in our patient cytological examination of the cyst fluid had not shown suspicious cells. Retrospectively, the brown colour of the cyst fluid which was also observed by Bloustein[3] as in cases of adenocarcinoma arising from non-parasitic hepatic cysts[13,14] in combination with the unusual hyperechoic pattern of cyst content in sonography might be considered as a sign for cyst atypia. The extreme elevation of erythrocyte sedimentation rate was also remarkable and could not be explained by another finding elsewhere.

**Table 1** Squamous Cell Carcinoma from Solitary Non-Parasitic Hepatic Cyst – Review of the Literature

| *Author and year* | *Age Sex Therapy* | *Survival* |
|---|---|---|
| Edmondson, 1958 | 56 M unknown | unknown |
| Greenwood *et al.* 1972 | 37 M expl.lap. | 2mo |
| Bloustein *et al.* 1976 | 30 M 1. surg. drain<br>2. Roux-en-Y drain.<br>3. extend. right hemihep.<br>4. expl. lap | 6mo |
| Sanz-Esponera *et al.* 1979 | unknown | unknown |
| Gresham *et al.* 1985 | 78 M none | 2mo |
| Lynch *et al.* 1988 | 63 M 1.unroofing<br>2. subtot. cystect. | 6mo |
| Nieweg *et al.* 1992 | 62 expl. lap. | 5mo |
| Pliskin *et al.* 1992 | 82 M none | 13d |
| Banbury *et al.* 1994 | 59 tight lobectomy | 16mo |

Modified from Lynch *et al.* 1988 (6)

*Treatment and prognosis*

Radical treatment with partial hepatectomy could be performed in Bloustein's[3], Banbury's[9] and our case. Bloustein's[3] patient died four months after resection with known tumor recurrence confirmed by exploratory laparotomy, the patient of Banbury[9] has been alive for 16 months without evidence for disease. In the other cases [2,5,6,8] due to central bilobar or advanced tumor no resection for cure could be performed-one patient was considered for liver transplantation. All these patients died within two weeks and three months after diagnosis. According to these findings prognosis of squamous cell carcinoma is unfavourable, once the tumor is beyond the cystic wall and infiltrating liver parenchyma.

There are no sharp guidelines for the treatment of solitary liver cysts. Asymptomatic cysts are an incidental finding in sonography or in CT-scan. Before presuming the diagnosis of congenital non-parasitic cyst, it is our opinion that Echinococcus disease should be excluded. Asymptomatic nonparasitic cysts do not require any treatment[23]. However, these cysts should be observed by sonography to assess possible growth. As in the presented case report, symptomatic cysts can be treated conservatively by sonography guided cyst drainage-larger ones by a pigtail catheter for one or two days-followed by sclerotherapy with ethoxysclerol. Therapy is successful in about 80% by single treatment[24]. Despite the rare occurence of carcinoma originating from a non-parasitic cyst, with regard to the problems of diagnosis and the unfavourable prognosis, an aggressive surgical approach can be considered: After twice failure of conservative therapy in a three months period, especially in case of brown cyst fluid, exploratory laparotomy or laparoscopy should be suggested for cyst unroofing and multiple biopsies with frozen sections from the cyst wall including the ground. Tumor positive biopsy should be followed by partial hepatectomy. In case of negative histology and postoperative cyst recurrence after unroofing partial hepatectomy can be also considered.

### REFERENCES

1. Edmondson H.A.(1958) Tumors of the liver and intrahepatic bile ducts. In: Edmondson. H.A, ed. Atlas of tumor pathology, edited by H.A. Edmondson, sec 7, fascicle 25. First series, pp 109–110. Washington DC: Armed Forces Institute of Pathology
2. Greenwood N., Orr W.M. (1972) Primary squamous-cell carcinoma arising in a solitary non-parasitic cyst of the liver. *J. Pathol.*, **107**, 145–8
3. Bloustein P.A.,Silverberg S.G.(1976) Squamous cell carcinoma originating in an hepatic cyst-case report and review of the hepatic cyst-carcinoma association. *Cancer*, **38**, 2002–2005
4. Sanz Esponera J., Castiella Muruzabal T., Lazaro Perez J. (1979) Squamous cell carcinoma of the liver developed in a simple nonparasitic cyst. *Pathology*, **165**, 158 (abstr)
5. Gresham G.A.,Rue L.W. 3d. (1985) Squamous cell carcinoma of the liver. *Hum. Pathol.*,**16**, 413–416.
6. Lynch M.J., Mc Leod M.K., Weatherbee L., Gilsdorf J.R., Giuce K.S., Eckhauser F.E.(1988) Squamous cell cancer of the liver arising from a solitary benign nonparasitic cyst. *Am.J.Gastroenterol.*, **83**, 426–431
7. Nieweg O., Sloof M.J.H., Grond J. (1992) Case Report – a case of primary squamous cell carcinoma of the liver arising in a solitary cyst. *HPB Surgery*, **5**: 203–208.
8. Pliskin A., Cualing H., Stenger R.J.(1992) Primary squamous cell carcinoma originating in congenital cysts of the liver-report of a case and review of the literature. *Arch. Lab. Med.*, **116**, 105–107
9. Banbury J., Conlon K.C., Ghossein R., Brennan M.F. (1994) Primary squamous cell carcinoma within a solitary nonparasitic hepatic cyst. *J. Surg. Oncol.*, **57**: 210–212.
10. Willis R.A.(1943) Carcinoma arising in congenital cysts of the liver. *J. Pathol.*, **55**, 492–495
11. Richmond H.G.(1956) Carcinoma arising in congenital cysts of the liver. *J. Pathol.*, **72**, 681–684
12. Dean D., Bauer H.M.(1969) Primary cystic carcinoma of the liver. *Am. J. Surg.*, **117**, 416–420
13. Ameriks J., Appleman H., Frey Ch. (1972) Malignant nonparasitic cyst of the liver. *Ann. Surg.*, **176**, 713–717
14. Kasai Y., Sasaki E., Tamaki A., Koshino I., Kawanishi N., Hata Y. (1977) Carcinoma arising in the cyst of the liver-report of three cases. *Jpn. J. Surg.*, **7**, 65–72
15. Mizumoto R.,Kawarada Y..(1987) Diagnosis and treatment of cholangiocarcinoma and cystic adenocarcinoma of the liver. In Neoplasms of the liver, edited by K. Okuda and K.G. Ishak, pp. 381–396, Tokyo, Berlin Heidelberg, New York, London, Paris:Springer
16. Ishak K.G., Willis G.W., Cummins S.D.,Bullock A.A (1977) Biliary cystadenoma and cystadenocarcinoma-report of 14 cases and review of the literature. *Cancer*, **38**, 322–338
17. Azizah N., Paradinas F.J. (1980) Cholangiocarcinoma coexisting with developmental liver cysts: a distinct entity different from liver cystadenocarcinoma. *Histopathology*, **4**, 391–400
18. Song E., Kew M.C. Grieve T., Isaacson C., Myburgh J.A.(1984) Primary squamous cell carcinoma of the liver occuring in association with hepatolithiasis. Cancer, **53**, 542–546
19. Clements D., Newman P., Etherington R., Lawrie B.W., Rhodes J. (1990) Squamous carcinoma in the liver. *Gut*, 31, 1333–1334
20. Barr R.J.,Hancock D.E.(1975) Adenosquamous carcinoma of the liver. *Gastroenterology*, **69**, 1326–1330
21. Nakjima T., Kondo Y. (1990) A clinicopathologic study of intrahepatic cholangiocarcinoma containing a component of squamous cell carcinoma. *Cancer*, **65**, 1401–1404
22. Clark D.D., Marks Ch., Bernhard VM., Bunkfeldt Jr F. (1967) Solitary hepatic cysts. *Surgery*, **61**, 687–93
23. Longmire W.P.Jr, Mandiola S.A., Goron H.E.(1971) Congenital cystic disease of the liver and the biliary system. *Ann. Surg.*, **174**, 711–726
24. Gebel M., Schulz M., Martin St.(1988) Short and long term results of ultrasonically guided therapy of non-parasitic liver cysts. *J. Ultrasound Med.*, **7**, S202(abstr)

